# Abnormal Blood Bacteriome, Gut Dysbiosis, and Progression to Severe Dengue Disease

**DOI:** 10.3389/fcimb.2022.890817

**Published:** 2022-06-17

**Authors:** Wiwat Chancharoenthana, Supitcha Kamolratanakul, Wassawon Ariyanon, Vipa Thanachartwet, Weerapong Phumratanaprapin, Polrat Wilairatana, Asada Leelahavanichkul

**Affiliations:** ^1^ Department of Clinical Tropical Medicine, Faculty of Tropical Medicine, Mahidol University, Bangkok, Thailand; ^2^ Tropical Immunology and Translational Research Unit, Department of Clinical Tropical Medicine, Faculty of Tropical Medicine, Mahidol University, Bangkok, Thailand; ^3^ Cardiometabolic Centre, Department of Medicine, Bangkok Nursing Hospital, Bangkok, Thailand; ^4^ Department of Medicine, Banphaeo General Hospital, Samutsakhon, Thailand; ^5^ Immunology Unit, Department of Microbiology, Faculty of Medicine, Chulalongkorn University, Bangkok, Thailand; ^6^ Center of Excellence on Translational Research in Inflammation and Immunology (CETRII), Department of Microbiology, Chulalongkorn University, Bangkok, Thailand

**Keywords:** dengue infection, immunity, leaky gut syndrome, lipopolysaccaride, beta-D-glucan, microbiome & dysbiosis

## Abstract

Despite a well-known association between gut barrier defect (leaky gut) and several diseases, data on translocation of pathogen molecules, including bacterial DNA (blood bacteriome), lipopolysaccharide (LPS), and serum (1→3)-β-D-glucan (BG), from the gut to the blood circulation (gut translocation) in dengue are still less studied. Perhaps, dengue infection might induce gut translocation of several pathogenic molecules that affect the disease severity. At the enrollment, there were 31 dengue cases in febrile and critical phases at 4.1 ± 0.3 days and 6.4 ± 1.1 days of illness, respectively, with the leaky gut as indicated by positive lactulose-to-mannitol excretion ratio. With blood bacteriome, the patients with critical phase (more severe dengue; n = 23) demonstrated more predominant abundance in Bacteroidetes and *Escherichia* spp. with the lower Bifidobacteria when compared with the healthy control (n = 5). Meanwhile, most of the blood bacteriome results in dengue with febrile stage (n = 8) were comparable to the control, except for the lower Bifidobacteria in dengue cases. Additionally, endotoxemia at the enrollment was demonstrated in five (62.5%) and 19 (82.6%) patients with febrile and critical phases, respectively, while serum BG was detectable in two (25%) and 20 (87%) patients with febrile and critical phases, respectively. There were higher peripheral blood non-classical monocytes and natural killer cells (NK cells) at the enrollment in patients with febrile phage than in the cases with critical stage. Then, non-classical monocytes (CD14^-^CD16^+^) and NK cells (CD56^+^CD16^-^) increased at 4 and 7 days of illness in the cases with critical and febrile stages, respectively, the elevation of LPS and/or BG in serum on day 7 was also associated with the increase in monocytes, NK cells, and cytotoxic T cells. In summary, enhanced Proteobacteria (pathogenic bacteria from blood bacteriomes) along with increased endotoxemia and serum BG (leaky gut syndrome) might be collaborated with the impaired microbial control (lower non-classical monocytes and NK cells) in the critical cases and causing more severe disease of dengue infection.

## Introduction

Dengue infection is a mosquito-borne infectious disease, causing a wide spectrum syndrome ranging from benign febrile illness to serious multiple organ failure, also known as severe dengue infection (World Health Organization, 2019). The progression of dengue infection to severe dengue commonly occurs after the febrile phase–between days 3 and 7 from the onset (World Health Organization, 2022). Then, the warning signs that might be associated with the development of severe dengue infection are the parameters of vascular permeability and plasma leakage (Halstead & Cohen, 2015). The impact of adaptive immunity in dengue is well known as a mismatch between the infecting viral serotype and the memory adaptive immunity which exacerbates dengue infection, termed antibody-dependent enhancement (ADE; the interaction between Fc domain of immunoglobulin G (IgG) and Fcγ receptors (FcγRs) on leukocytes (Bournazos et al., 2020) that enhances FcγRs-mediated viral entry mechanism). Accordingly, circulating immune cells and soluble immune components can be detected in peripheral blood and may serve as prognostic markers as a result of the direct consequence of dengue infection.

In contrast, data on the influence of innate immunity in dengue infection are still less. It has recently been suggested that lipopolysaccharide (LPS) (also known as endotoxins) and (1→3)-β-D-glucan (BG) in serum are possibly transferred from the gut (leaky gut syndrome or gut translocation) and may play a major role in the severity of dengue infection through the LPS and BG pro-inflammatory properties, especially through innate immune responses. Serum LPS and BG are proposed as the new prognostic factors at admission to identify dengue patients with poor outcomes (Chancharoenthana et al., 2021). Both LPS and BG are the major cell wall components of Gram-negative bacteria and fungi, respectively, which are the most and second most abundant of organisms in the human gut, respectively (Lagier et al., 2012). In the healthy gut permeability, there is a limited gut translocation of both LPS and BG, partly because the size of these molecules (50-100 kDa) is too large to pass through the normal intestinal tight junction (Benoit et al., 1998; Finkelman, 2020). The injury of the gut tight junction by several causes, including severe dengue infection, induces gut translocation of these molecules allowing the use of these molecules as the indirect biomarkers for gut barrier defect. Moreover, both LPS and BG are foreign microbial molecules for the mammalian host, referred to as “pathogen-associated molecular patterns (PAMPs),” which profoundly activate innate immunity and facilitate adaptive immune responses through several immune cells (Isnard et al., 2021; Tiku & Tan, 2021). Not only LPS and BG, but gut bacterial DNA are also PAMPs with possible gut translocation during leaky gut with a property of innate immunity activation. Although intact bacterial DNAs (genomes) with molecular sizes of 100 to 15,000 kbp (6.5x10^4^–9.8x10^6^ kDa) cannot pass through the gut barrier, bacteria-free DNA is rapidly naturally broken down into smaller sizes of less than 100 bp (lower than 65 kDa) by several processes (depurination or deamination DNA), which can pass across the gut barrier in numerous models. Moreover, exploration of the bacterial genome in the blood (blood bacteriome) are also crudely associated with an alteration in fecal bacteriome (gut dysbiosis) (Kaewduangduen et al., 2022) that might be correlated with the severity of dengue infection as gut dysbiosis between sepsis and non-sepsis are different (Panpetch et al., 2018; Amornphimoltham et al., 2019). Hence, the different patterns of blood bacteriome might be used as another parameter for determining dengue severity.

The early identification of patients with poor prognoses would help physicians provide the proper therapeutic management, and the associated parameters of immune response variability might determine disease severity. Despite the current proposed dengue prognostic parameters, including C-reactive protein polymorphism variants (Mukherjee & Tripathi, 2020), ferritin (Suresh et al., 2020), granulocyte-macrophage colony-stimulating factor (GM-CSF) (Lee et al., 2019), interferon gamma-inducible protein-10 (IP-10) (Chen et al., 2006), and vascular injury biomarkers (Vuong et al., 2021), leaky gut-associated parameters and innate immunity determination are also interesting. Furthermore, very little is known about the physiologic consequences of leaky gut, blood bacteriome, and gut dysbiosis in dengue as host-microbiome during dengue infection which can be beneficial or harmful to the host (González & Elena, 2021). Here, the prospective single-center analysis on patients with dengue infection was conducted to see if the biomarkers of gut translocation, bacteriomes, and innate responses could be used as the prognostic factors in dengue infection. Targeting gut barrier dysfunction and reducing pathogen translocation are emerging strategies for the prevention and treatment of severe dengue infection (Chancharoenthana et al., 2021) and may be useful in other inflammatory diseases.

## Materials and Methods

### Enrolled Participants and Study Designs

Samples were collected during the outbreak of dengue between November 2020 and November 2021. Dengue patients diagnosed by the NS1 antigen test (Platelia enzyme-linked immunosorbent assay (ELSA); Bio-Rad) and commercial immunoglobulin (Ig) M and IgG serology assays (Capture ELISA; Panbio) during the febrile or critical phase were recruited. Reverse-transcription polymerase chain reaction (RT–PCR) was also performed to identify the viral serotype. The dengue-confirmed cases were defined if the RT–PCR, NS1 antigen, or IgM assays were positive at enrollment or if there was IgM seroconversion between the paired specimens, following World Health Organization (WHO, Geneva, Switzerland) criteria (World Health Organization, 2009). The severity of dengue infection was assessed based on their clinical spectrum as modified from the 2009 WHO guideline for dengue infection as follows (World Health Organization, 2009): the febrile phase of dengue infection refers to the 1st through 3rd days of illness whereas the critical phase is the time between the 4th and 7th days of illness. Although, the 2009 WHO guideline identifies febrile phase as the 2nd through 7th^t^ days after onset, most cases in our experience demonstrate the febrile phase only between 1st through 3rd days of the illness similar to other studies (Yacoub et al., 2014; Simmons et al., 2015). Meanwhile, the warning signs refer to clinical symptoms of abdominal pain or tenderness, persistent vomiting, as well as clinical signs of fluid accumulation, mucosal bleeding, lethargy, hepatomegaly, and hemoconcentration in combination with thrombocytopenia. Patients with at least one of the following symptoms, severe plasma leakage, severe bleeding, or severe organ involvement, were classified as severe dengue infection (World Health Organization, 2009) and patients 18 to 50 years old were defined as eligible participants. Then, the participants were first tested for intestinal permeability with the urinary lactulose-to-mannitol excretion ratio (LMER) test as previously described. Briefly, the mixture of lactulose and mannitol consisting of 5 g lactulose, 2 g mannitol, and 22.3 g glucose dissolved in 100 mL sterile water with osmolarity at 1500 mOsmol/L was orally administered. Then, the LMER was assessed by isocratic ion-exchange high-performance liquid chromatography (HPLC) with mass spectrometry (Thermo Fischer Scientific, Waltham, MA) to determine the concentrations of lactulose and mannitol in the urine samples. Participants with positive LMER or ratio > 0.1178 were defined as positive (impaired) intestinal permeability (Chancharoenthana et al., 2021). In addition, regarding the LMER test, participants with the following conditions were excluded: (1) chronic gastrointestinal diseases, (2) history of gastrointestinal surgery, (3) familial history of inflammatory/irritable bowel syndrome, (4) known to hypersensitivity reaction to mannitol and/or lactulose, (5) metabolic syndrome-related conditions, (6) autoimmune disease, and (7) presence of alcohol intake or substance misuse or recent use of medication besides acetaminophen or probiotic. Blood specimens were collected in heparinized tubes for studies. The present prospective cohort study was approved by the Ethics Committee of the Faculty of Tropical Medicine, Mahidol University (MUTM 2020-071-01), and registered in the Thai Clinical Trials Registry (TCTR20210208002) in accordance with STROBE guidelines. Written informed consent was obtained from all participants.

### Blood Microbiome Study

**Figure 1:**
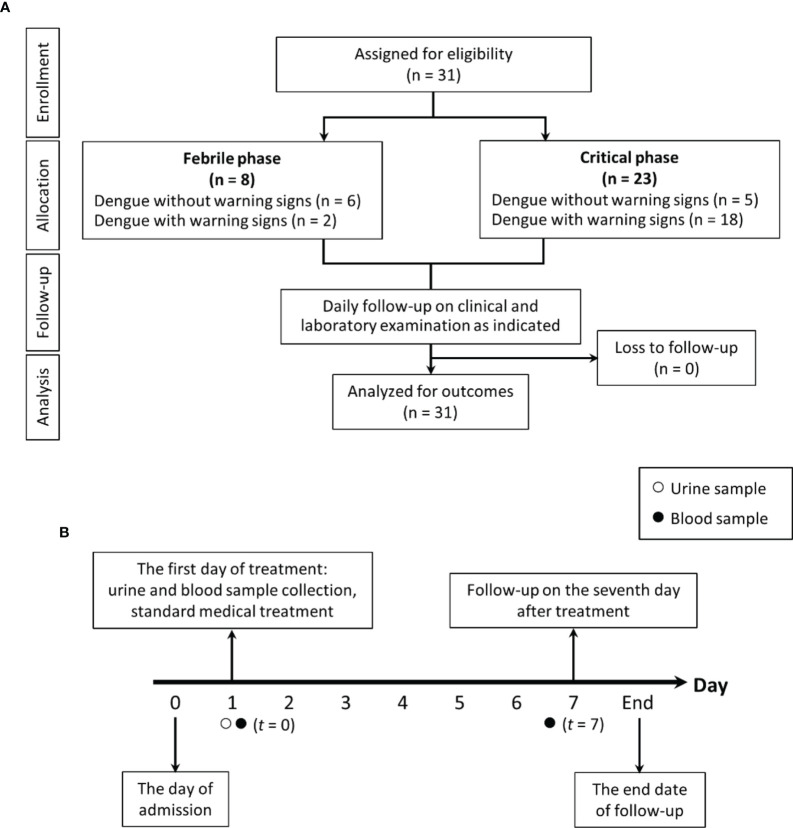
The schematic diagram demonstrates for study flow diagram **(A)** and timeline of the study design **(B)**.

Blood microbiome study was tested in all participants once enrolled (*t* = 0) (**Figure 1**). In brief, genomic DNA was extracted from 300 µl of whole blood using the GenUp™ gDNA kit (Biotechrabbit, Germany). The amplification of the bacterial 16S rDNA was performed in total volume 25 µl consisting of *Taq* DNA polymerase (0.5U) (Biotechrabbit, Germany), 1.5 mM MgCl_2_, 0.2 mM dNTPs, 0.2 mM forward primer 5′-ACTCCTACGGRAGGCAGCAG-3′, and 0.2 mM reverse primer 5′-CCGTCAATTYYTTTRAGTTT-3′. The PCR product was re-amplified within V4 region of 16S rDNA by using phasing adaptor primers following from Wu and colleagues (Wu et al., 2015). Amplified PCR products (~ 430 bp) were purified by using the QIAquick PCR Purification Kit (QIAGEN, Germany) and quantified by KAPA library quantification kits for Illumina platforms (Kapa Biosystems, Wilminton, MA, USA). The DNA libraries were pooled at equal amount and paired-end (2 × 250 cycles) sequenced on an Illumina MiSeq platform with a MiSeq Reagent Kit V2 (Illumina, USA).

### Flow Cytometry for Immunity Parameters

The identification of different cell types was performed by the flow cytometry analysis using AQUIOS CL with several antibodies (Beckman Coulter, Brea, CA, USA) by the central laboratory of the Faculty of Tropical Medicine, Mahidol University, as previously described (Gossez et al., 2017). Briefly, 50-μL of blood were stained with anti-CD45-fluorescein isocyanate (FITC), the lymphocyte common antigen, before identification as several lymphocytes using anti-CD3-proprotein convertase 5 (PC5), anti-CD4-RD1, anti-CD8-Ecdysoneless Homolog (Drosophila) (ECD), and anti-CD-19-allophycocyanin (APC). Likewise, the classical and intermediated monocytes were indicated by CD14^+^/CD16^-^ and CD14^+^CD16^+^, respectively, while the non-classical monocytes were CD14^-^/CD16^+^. In parallel, natural killer (NK) cells were indicated by CD56^+^CD16^-^ cells. Then, the samples were run on a flow cytometry.

### Serum and Blood Parameters

Samples were used to measure interleukin-6 (IL-6) cytokine using ELISA (Enzo Life Sciences, Inc., Farmingdale, NY, USA). Serum (1→3)-β-D-glucan (BG) was measured by a Fungitell^®^ assay (Associates of Cape Cod, Falmouth, MA). The Fungitell^®^ assay detects BG through activation of factor G, a protease zymogen, which cleaves a chromophore from a chromogenic peptide through light absorbance at 405 nm following the manufacturer protocol. Briefly, 5 mL of sample was mixed with an alkaline pretreatment reagent (0.125 M KOK/0.6 M KCl) and incubated at 37°C for 10 min, and the reconstituted Fungitell reagent (100 µL) was added to each well before measurement. The detectable range of BG was 7.8–523.4 pg/mL, thus, BG values of < 7.8 pg/mL and > 523.4 pg/mL were recorded as 0 and 523 pg/mL, respectively. In parallel, lipopolysaccharide (LPS) in blood was assessed by chemiluminescence-based endotoxin activity (EA) assay (Spectral Diagnostics, Toronto, ON, Canada) based on the detection of enhanced respiratory burst activity in neutrophils following their priming by complexes of endotoxin and the specific anti-endotoxin antibody (Marshall et al., 2004). Briefly, 50 μL of whole blood was incubated in duplicate with saturating concentrations of an anti-lipid A IgM antibody and stimulated with opsonized zymosan, and respiratory burst activity was detected from the lumiphor luminol by a chemiluminometer (Berthold Technologies, Bad Wildbad, Germany). The endotoxin results in EU/mL were converted to pg/mL. EA levels of 0.4 and 0.6 are approximately equivalent to endotoxin concentrations of 500 and 1500 pg/mL, respectively. All samples were kept frozen at −80°C until analyses. Most of the parameters were measured on the enrollment date (*t =* 0) and on the 7th day of admission (*t* = 7), as shown in **Figure 1**. Additionally, all patients were clinically reviewed daily until fully recovered and afebrile or boffset="10"for up to 7 days after enrollment. The data analysis are based on characteristics of participants at the enrollment regardless of the duration after the disease onset. Five healthy control participants were included in the blood microbiome study with mean age 36.4 ± 8.2 years old. All of the healthy controls had no previous diagnosis of dengue fever; no recent use of medication, prebiotic, probiotic, or vitamin supplements; as well as no history of gastrointestinal surgery or chronic gastrointestinal disease.

### Statistical Analysis

Quantitative data are summarized as the mean and standard deviation (SD) for normally distributed variables or median (IQR) for nonnormally distributed variables, and qualitative data are presented as *n* (percentage). Normally distributed variables, non-normally distributed variables, and categorical variables were compared by Student’s *t*-test, Mann–Whitney U test, and chi-square test, respectively. Welch correction was also applied when appropriate. A *p* < 0.05 was considered statistically significant. Correlations were calculated using Pearson’s correlation coefficient. Logarithmic transformation was used if the data were not normally distributed. Principal component analysis (PCA) ordinations were used to visualize the clustering of immune cell profiles. Data analysis was performed using the PASW 18.0.0 statistical software package (SPSS Inc., Chicago, IL, USA) and GraphPad Prism 9.2.0 software (GraphPad Software, Inc., La Jolla, CA, USA).

## Results

### Baseline Characteristics of Participants

A total of 31 dengue inpatients with impaired intestinal permeability determined by the LMER test were analyzed. Of all patients, eight (25.8%) patients were in the febrile phase of dengue infection, whereas 23 (74.2%) patients were in the critical phase. Baseline characteristics in both groups of patients were similar in terms of demographics, gastrointestinal symptoms, and dengue serotypes (**Table 1**). At the time of enrollment, patients with dengue who were in the febrile phase had a lesser duration of illness, with an average of 4 days after fever, compared to those in the critical phase. Patients with dengue in the febrile phase also had higher hemoconcentration but lower lymphocyte counts compared with those in the critical phase. Using the 2009 WHO classification, there were two (25%) and 18 (78%) patients with dengue warning signs in the febrile and critical phases, respectively. As such, most of the participants were classified as dengue with warning signs. Among 31 patients with PCR positive for dengue, the individuals with dengue virus DENV-1 and DENV-2 serotypes were three (9.7%) and 24 (77.4%), respectively, while four patients (12.9%) had DENV-4. Of note, there was no significant difference between primary and secondary infection determined by serology assays using IgM and IgG, respectively, between dengue in the febrile phase versus the critical phase (**Table 1**). Additionally, all baseline intestinal permeability and inflammatory marker data were non-significantly different between these groups (**Table 1** **)**. Notably, the data analysis was performed at the recruitment regardless of the duration after the disease onset.

**Table 1:** Differences in baseline characteristics and clinical parameters between dengue patients in febrile phase and critical phase.

	Dengue in febrile phase (n = 8)	Dengue in critical phase (n = 23)	*P-value*
Age, years	38.7 ± 9.5	41.5 ± 8.2	0.431
Female, n (%)	5 (62.5)	14 (60.9)	0.937
Day of illness at enrollment	4.1 ± 0.3	6.4 ± 1.1	<0.0001
NS1 positive, n (%)	8 (100.0)	23 (100.0)	–
IgM positive, n (%)	8 (100.0)	23 (100.0)	-
IgG positive, n (%)	2 (25.0)	7 (30.4)	0.776
PCR positive, n (%)			
Serotype DENV-1	1 (12.5)	2 (8.7)	0.758
IgG positive	1 (12.5)	2 (8.7)	0.758
Serotype DENV-2	6 (75.0)	18 (78.3)	0.850
IgG positive	1 (12.5)	4 (17.4)	0.750
Serotype DENV-3	0 (0)	0 (0)	–
IgG positive	0 (0)	0 (0)	-
Serotype DENV-4	1 (12.5)	3 (13.0)	0.972
IgG positive	0 (0)	1 (4.3)	0.558
Symptoms, n (%)			
Fever	8 (100.0)	23 (100.0)	–
Anorexia	8 (100.0)	23 (100.0)	–
Nausea	8 (100.0)	23 (100.0)	–
Abdominal pain	2 (25.0)	16 (69.6)	0.030
Diarrhea	2 (25.0)	5 (21.7)	0.850
Myalgia	7 (87.5)	14 (60.9)	0.173
Rash	1 (12.5)	10 (43.5)	0.121
Mucosal bleeding	2 (25.0)	5 (21.7)	0.850
Clinical fluid accumulation	0 (0)	5 (21.7)	0.157
Hemoglobin (g/dL)	13.9 ± 1.9	11.6 ± 2.5	0.025
Hematocrit (%)	40.5 ± 3.4	34.1 ± 2.7	<0.0001
White blood cell count (x10^9^/L)	3.66 ± 1.4	5.68 ± 1.9	0.010
Lymphocytes (%)	49.3 ± 3.6	42.7 ± 2.2	<0.0001
Neutrophils (%)	47.2 ± 1.8	52.5 ± 0.9	<0.0001
Monocytes (%)	3.5 ± 0.2	4.8 ± 0.3	<0.0001
Platelet count (x10^9^/L)	174.8 ± 12.1	103.4 ± 18.9	<0.0001
Aspartate transaminase (AST) (U/L)	110.6 ± 14.1	168.8 ± 12.2	<0.0001
Alanine transaminase (ALT) (U/L)	53.6 ± 6.2	59.7 ± 11.8	0.176
LMER, median (range)	0.156 (0.128-0.256)	0.196 (0.134-0.267)	0.288
Endotoxins (pg/mL)	308.3 ± 188.9	359.0 ± 113.1	0.369
(1→3)-β-D-glucan (BG) (pg/mL)	117.1 ± 59.4	134.8 ± 63.1	0.494
Interleukin (IL)-6 (pg/mL)	408.0 ± 203.5	322.7 ± 146.3	0.210

LMER, lactulose-to-mannitol excretion ratio; PCR, polymerase-chain reaction.

### The Progression to Severe Dengue Infection as Stratified by Warning Signs of Dengue Infection

On the 7th day of admission (t =7) (**Figure 1B**), there were 24 of 31 patients (77.4%) with severe dengue infection with the following characteristics: (i) severe plasma leakage, including shock (13 patients; 54.2%) and respiratory distress from fluid accumulation (four patients; 16.7%); (ii) severe bleeding (four patients;16.7%); and (iii) high aspartate transaminase (AST) >1,000 U/L (five patients; 20.8%). Among 24 patients with severe dengue as categorized by LMER and warning signs, there were 18 patients (75%) with positive LMER plus positive warning signs (two patients from febrile phase and 16 in critical phases of infection) and six patients (25%) with positive LMER without warning signs (three patients from febrile phase and another three patients from the critical phase).

### Differences of Blood Microbiome Spectrum Between Dengue Patients in Critical Phase and Febrile Phase

**Figure 2:**
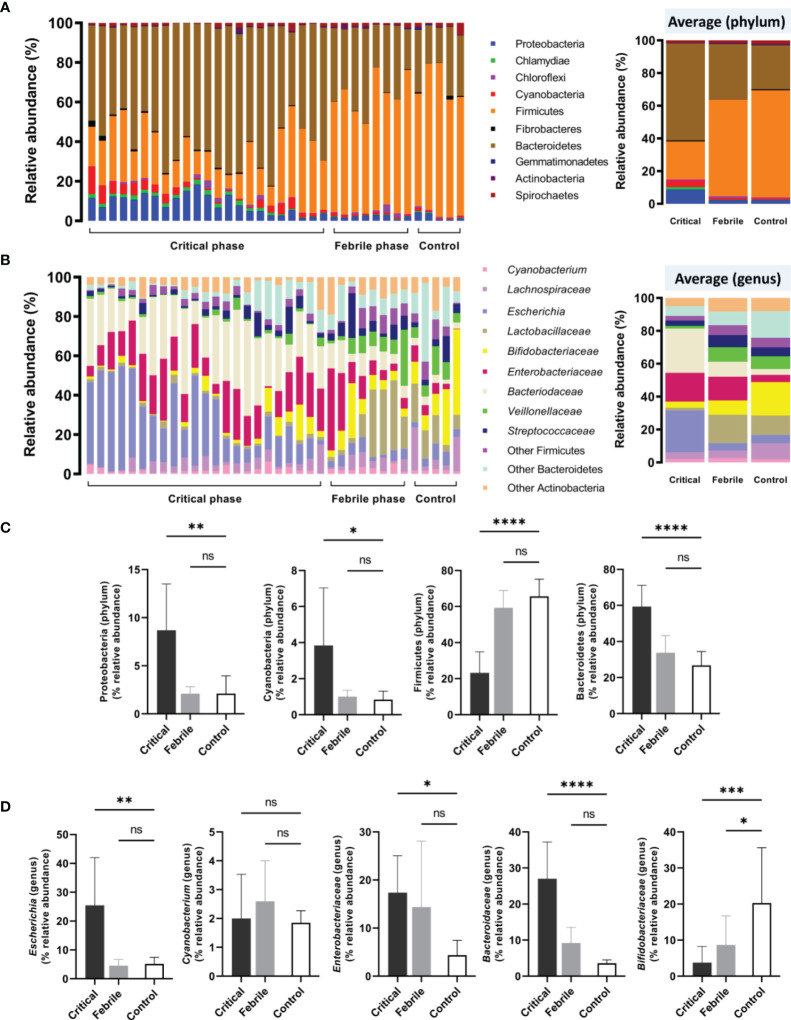
Blood microbiome analysis (blood bacteriome) from samples of dengue patients in critical phase (Critical, n = 23), febrile phase (Febrile, n = 8), and healthy controls (Control, n = 5) as indicated by **(A)** the phylum-level analysis (with average values), **(B)** genus-level analysis (with average values, and selected phylum **(C)** and genus **(D)** analysis are demonstrated. *p < 0.05; **p < 0.005; ***p < 0.0005; ****p < 0.0001; and ns, non-significant between the indicated groups using one-way ANOVA with Tukey’s analysis.

With gut barrier defect, bacterial DNA in the gut that is possibly associated with the abundance of intestinal bacteria can pass through the blood circulation (gut translocation) (Kaewduangduen et al., 2022). Indeed, blood bacteriome was different between the patients with critical phase and febrile stage (**Figure 2**), despite a similar impaired intestinal permeability (positive LMER) in all participants (**Table 1**). In the phylum level of analysis, patients with the critical phase demonstrated predominant Bacteroidetes (highest abundance Gram-negative anaerobes in the gut), Proteobacteria (mostly pathogenic Gram-negative aerobes), and Cyanobacteria (Gram-negative bacteria with possible toxins) with lower Firmicutes (mostly Gram-positive anaerobes that dominant in healthy conditions) (Panpetch et al., 2020; Thim-Uam et al., 2022) compared with the healthy control (**Figures 2A–C**). Meanwhile, blood bacteriome between patients with febrile stage and control was not different (**Figures 2A–C**) supporting gut translocation of bacterial DNA as possible normal physiology in healthy individuals (Camilleri, 2019) that was not altered by less severe dengue infection. In the genus level of analysis, *Bacteroidaceae* and *Escherichia* (Gram-negative bacteria with possible harmful impact) (Panpetch et al., 2020) in the critical phase dengue group were higher than control (**Figure 2D**), whereas the abundance of *Bifidobacteriaceae* (possible beneficial bacteria) (O’Neill et al., 2017; Panpetch et al., 2019) in healthy control was higher than in the dengue groups (both severity) (**Figure 2D**). Although fecal microbiome analysis of dengue patients was not explored, altered blood bacteriome in the infection groups compared with healthy control individuals implied gut dysbiosis during dengue infection. Patients who presented with abnormal blood bacteriome at enrollment had higher propensity to progress into severe dengue infection.

### Increased Lipopolysaccharides (LPS) and (1→3)-β-D-Glucan (BG) in Serum in Parallel With a Markedly Increased Monocytes, Natural Killer (NK) Cells, and Cytotoxic T Cells

**Figure 3:**
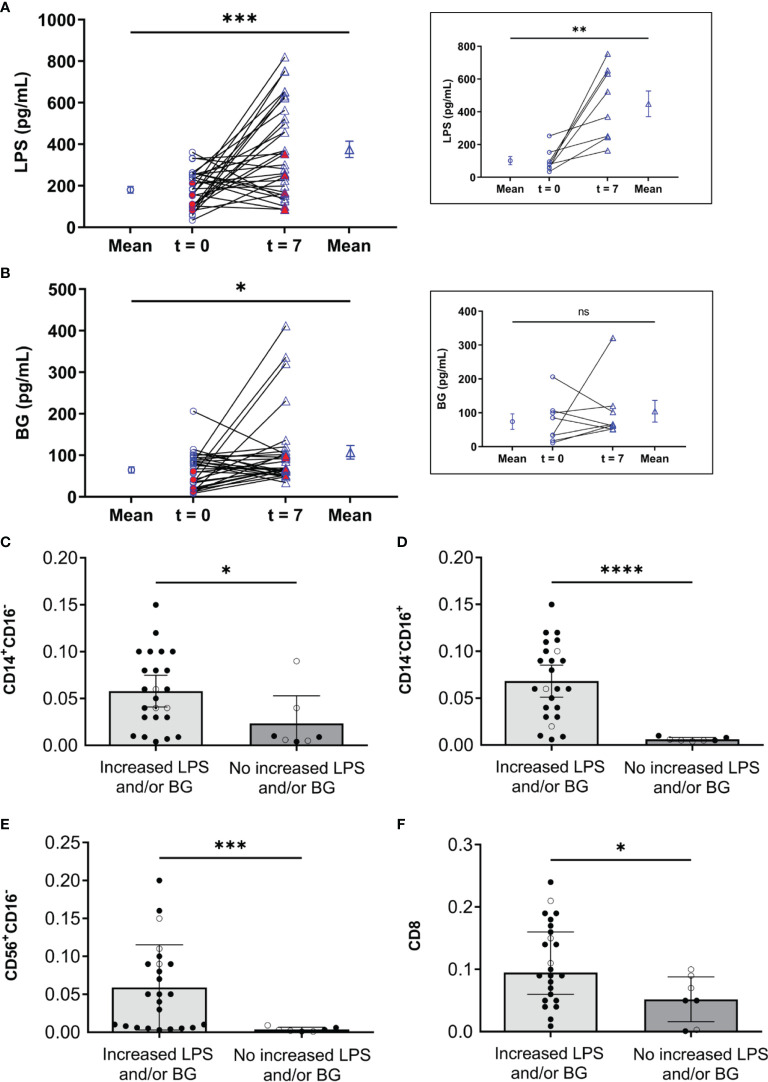
Roles of lipopolysaccharide (LPS), (1→3)-β-D-glucan (BG), and innate immunity parameters. **(A)** The difference of lipopolysaccharide (LPS) and **(B)** (1→3)-β-D-glucan (BG) between time enrollment (t = 0, ○) and day 7 of illness (t = 7, Δ) in dengue patients with impaired intestinal permeability (n = 31). The red dots in A and B represent patients with clinical severe dengue (plasma leakage, bleeding, or organ involvement; see method) during the observation. The inset boxes are value of LPS (inset box in A) and BG (inset box in B) in serum at t = 0 and t = 7 in patients who developed from febrile phase (1st through 3rd day of illness) to critical phase (4th through 7th day of illness) of dengue. Paired t-test was used for comparing mean. **(C, D)** blood abundance of monocytes (CD14^+^CD16^-^ and CD14^-^CD16^+^), **(E)** natural killer cells (CD56^+^CD16^-^), and **(F)** cytotoxic T cells (CD8) stratified by dengue patients with increased LPS and/or BG compared to those without increased LPS and/or BG. (C-F) ○ and ● in C-F refer to non-severe and severe dengue infection cases, respectively. Mann-Whitney U test was used for comparing median. ns, non-significant, *p < 0.05, **< 0.01, ***p < 0.0005, and ****p < 0.0001.

In gut barrier defect, not only bacterial DNA but also pathogen molecules in the gut (LPS and BG) are transferred from the gut into the blood circulation (Kaewduangduen et al., 2022). At enrollment, five (62.5%) and 19 (82.6%) patients with febrile and critical phases, respectively, had increased sequential LPS (mean difference 235.2 pg/mL (95% confidence interval (CI) 120.4 to 350.0), *p* = 0.0003) (**Figure 3A**) whereas two (25%) patients with febrile phase and 20 (87%) patients with critical phase at enrollment had increased sequential BG (mean difference 42.7 pg/mL (95% CI 4.65 to 80.65), *p* = 0.029) (**Figure 3B**). Additionally, there were eight patients who had a disease progression from febrile phase (1st through 3rd day of illness) on the admission day (t = 0; at the enrollment) into the critical phase (4th through 7th day of illness; see method) on day 7 of the admission (t = 7) (**Figure 3A**, inset box). On day 7 of the admission (t = 7), serum LPS, but not BG, of these eight patients with natural disease progression was increased when compared with the admission day (t = 0) (inset box of **Figures 3A, B**). Thus, the increased serum LPS might be associated with the natural progression from the febrile phase into the critical phase. On the other hand, during 7 days of the in-hospital observation, there were five patients out of eight patients with febrile phase at the enrollment who developed severe dengue (plasma leakage, bleeding, or organ involvement; see method) on day 7 of admission. Among these eight patients with severe dengue on day 7 of the admission, serum LPS and BG at the admission (t = 0) were not different from the levels on day 7 (red dots in **Figures 3A, B**). In parallel, there were 19 patients out of 23 patients with critical phase at the enrollment (4th through 7th day of illness) who had increased LPS and BG on day 7 of admission (t = 7) compared with the admission day (t =0) without the progression into severe dengue (plasma leakage, bleeding, or organ involvement; see method) (most of the empty dots in **Figures 3A, B**). Among 24 patients with increased LPS and/or BG, there were only three patients with severe dengue (empty dots in **Figures 3C–F**). Meanwhile, from seven patients with no elevation of LPS or BG, there were four patients who developed severe dengue (empty dots in **Figures 3C–F**). Then, serum LPS and BG levels could not be used for the prediction of the progression into severe dengue. Although serum LPS and BG between patients with febrile and critical phase on day 1 (t = 0) were not different (**Table 1**), patients with elevated LPS or BG in serum on day 7 of illness showed a significant increase in monocyte, NK cell, and CD8 T cell lineages compared with those without LPS and BG elevation (**Figures 3C–F**). Hence, the elevation of LPS and/or BG in dengue might be associated with the innate immune responses despite the non-correlation with severe dengue.

In parallel, dengue patients with impaired intestinal permeability and critical phase presentation showed increased and reduced percentages of peripheral blood neutrophils and lymphocytes, respectively (**Table 1**). To avoid skew interpretation in absolute lymphocyte counts, relative frequencies were further assessed. The higher ratio of CD4/CD8 was found in dengue patients with critical phase presentation compared with dengue patients in the febrile phase (2.33 vs. 1.92, respectively, *p* = 0.031), as shown in **Table 2**. On the other hand, both absolute CD4 and CD8 counts were significantly increased in dengue patients with the febrile phase. A significant increase in the percentage of non-classical monocytes, as well as NK cells (CD56^+^CD16^-^), were observed in the febrile group (see **Table 2**). Notably, no difference was observed in the frequency of B lymphocytes as determined by CD19 between dengue in the febrile phase and in the critical phase (**Table 2**).

**Table 2:** Comparisons of innate immunity parameters between dengue patients in febrile phase and critical phase.

	Dengue in febrile phase (n = 8)	Dengue in critical phase (n = 23)	*P-value*
%CD3	71.3 ± 12.2	60.9 ± 11.7	0.041
%CD4	45.1 ± 11.4	40.5 ± 10.9	0.318
%CD8	23.8 ± 6.2	18.2 ± 5.7	0.026
%CD4 to %CD8 ratio	1.92 (1.49-3.57)	2.33 (1.36-3.92)	0.031
%CD19	11.9 ± 6.7	12.8 ± 7.5	0.767
Absolute lymphocytes	1204 (812-2016)	892 (667-1321)	0.001
Absolute CD3	902 (508-1327)	609 (358-862)	<0.001
Absolute CD4	547 (353-867)	355 (221-504)	<0.001
Absolute CD8	220 (126-470)	153 (84-291)	0.012
Absolute CD19	111 (63-207)	91 (45-133)	0.652
Monocytes			
Classical (%CD14^+^CD16^-^)	70.34 (62.5-79.6)	71.11(49.5-82.2)	0.896
Intermediate (%CD14^+^CD16^+^)	27.9 (17.4-39.9)	27.7 (15.2-42.9)	0.677
Non-classical (%CD14^-^CD16^+^)	3.4 (1.2-6.6)	1.5 (0.6-3.5)	0.010
Natural killer (NK) cells			
% CD56^+^CD16^-^	4.2 (1.5-9.6)	3.2 (2.1-5.0)	0.034
Absolute CD56^+^CD16^-^(cells/μL)	169 (114-277)	159 (98-230)	0.205

For parametric and non-parametric variables, mean ± standard deviation (SD) and median (interquartile range) are shown.

**Figure 4:**
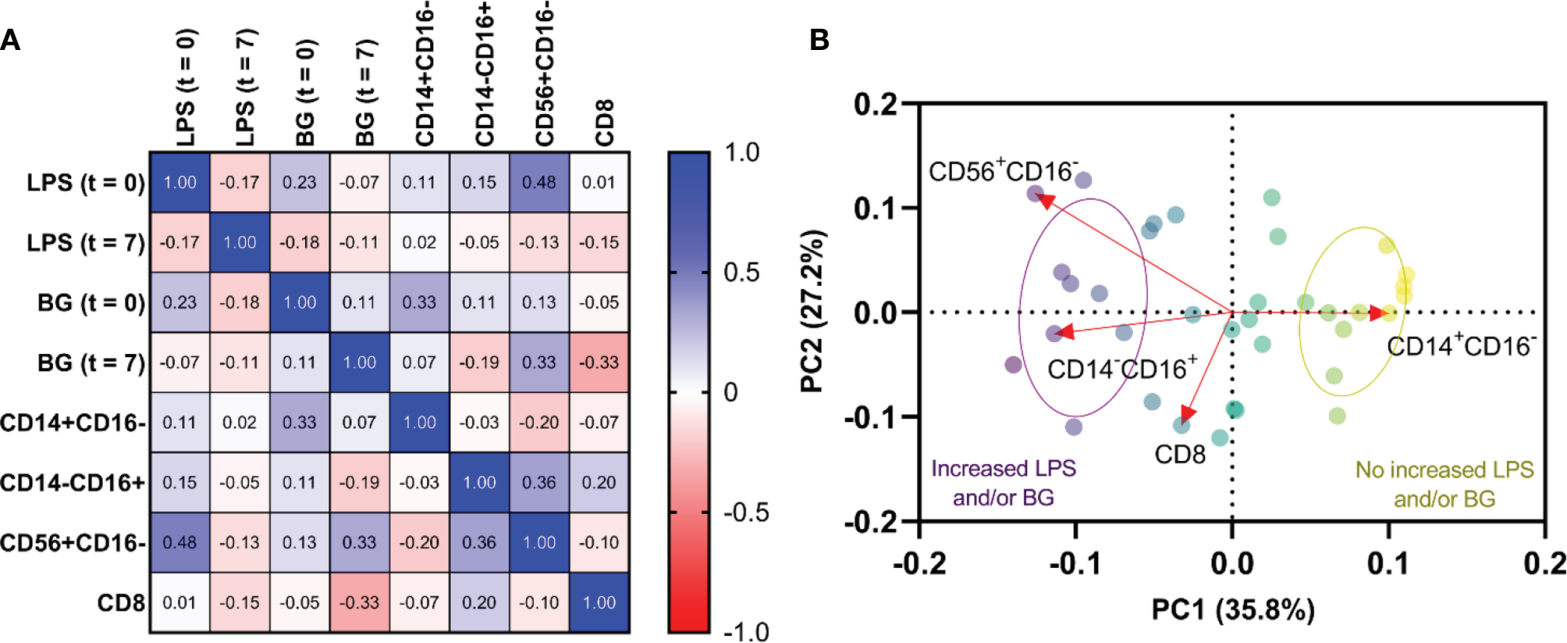
Relationship among lipopolysaccharide (LPS), (1→3)-β-D-glucan (BG), and immune profiles. Correlations of LPS and BG among monocytes(CD14^+^CD16^-^ and CD14^-^CD16^+^), natural killer (NK) cells (CD56^+^CD16^-^), and cytotoxic T cells (CD8^+^) at time of enrollment (*t* = 0) and day 7 of illness (*t* = 7) **(A)**. Principal component analysis of immune cells with increased LPS and/or BG and without increased LPS and/or BG **(B)**. Red arrows represent gradients of the corresponding immune cells and point to the direction of greatest increase in these measures. Color of the circles represents the combination of LPS and/or BG subgroups, and ellipses represent standard deviation (SD) of the groups.

Because the immune responses against LPS and BG in serum might alter several immune cells (monocytes, NK cells, and cytotoxic T cells), the correlation between LPS and BG in serum versus these cells was explored. Accordingly, both serum LPS and BG showed a positive correlation with increased monocytes and NK cells. As such, the NK cells (CD56^+^CD16^-^) showed a positive correlation to serum LPS at the time of enrollment (t = 0) (r = 0.479, *p* = 0.006), whereas non-classical monocytes (CD14^-^CD16^+^) had a tendency of positive correlation to BG at time of enrollment (t = 0) (r = 0.327, *p* = 0.07) (**Figure 4A**). PCA visualizations of the immune cell composition in the cohort of 31 dengue patients with impaired intestinal permeability revealed an opposite effect in those with versus without increased LPS and/or BG, indicating a stratification of the association between immune cell composition and these microbial molecules (LPS and/or BG) (**Figure 4B**).

## Discussion

Here, intestinal permeability impairment (leaky gut) in dengue patients was associated with blood bacteriome alteration and elevated pathogen molecules, lipopolysaccharide (LPS), and (1→3)-β-D-glucan (BG), in serum that activated several immune cells, especially the non-classical monocytes and natural killer (NK) cells.

Although dengue virus (DENV) causes a mild self-limiting illness in the majority of individuals, many cases are annually reported as severe dengue (World Health Organization, 2022) that, at least in some cases, is severe enough to cause leaky gut syndrome with translocation of pathogen molecules from the gut into the blood circulation (gut translocation). Because there might also be some direct impacts of DENV on enterocytes as DENV-V2 could directly infect Caco-2 cells, an intestinal cell line (Kar et al., 2019), the studies on intestinal aspects in patients with dengue are interesting. There was no correlation between dengue severity, as determined by endothelial leakage and bleeding tendency, and serotype of DENV (data not shown). Here, we demonstrated gut barrier defect and gut dysbiosis through several parameters (endotoxemia, serum BG, and LMER) and blood bacteriome, respectively. Gut barrier defect in systemic infections, including viral infection (such as COVID-19) (Sirivongrangson et al., 2020; Saithong et al., 2022) or viral diarrhea (such as Rota viral infection) (Warfield et al., 2006) is previously mentioned. The translocation of pathogen molecules from gut into blood circulation (gut translocation) affects immune responses in several condition (Panpetch et al., 2018; Amornphimoltham et al., 2019). During dengue-induced gut translocation, systemic inflammatory responses against these pathogen molecules (bacterial DNA, LPS, and BG) activates antigen-presenting cells, NK cells, T cells, and B cells (Fasano, 2011), induces profound pro-inflammatory cytokines, and enhances more severe gut translocation, including the viable organisms in some situations with severe gut barrier defect (Elinav et al., 2011; Leelahavanichkul et al., 2016a). Although the high level of LPS in serum induces several acute co-morbidities (mostly the systemic inflammation) through the activation of other immune cells (such as neutrophil extracellular traps) (Saithong et al., 2022) or the presence of other organelles in the blood (such as mitochondrial DNA) (Kaewduangduen et al., 2022), these parameters are not explored in the current study. More studies are needed.

Here, in dengue infection, our previous study has highlighted the importance of enhanced circulating LPS and BG as biomarkers of impaired intestinal permeability in comparison with lactulose-to-mannitol excretion ratio (LMER) (a standard biomarker), which might be associated with the severity of dengue infection (Chancharoenthana et al., 2021). In the present study, we focused on dengue patients with positive LMER test in either acute febrile phase or critical phase regardless of dengue severity. Several inflammatory mediators (Fasano & Shea-Donohue, 2005) along with prominent alteration in circulating bacteriome (Mu et al., 2017) were demonstrated in patients with gut barrier defects. The correlation among LMER, endotoxemia, and serum BG for the determination of gut barrier defects are previously mentioned (Chancharoenthana et al., 2021). Although several characteristics in the critical phase (plasma leakage and bleeding tendency) seem to be more severe than in the febrile phase, systemic inflammation (serum IL-6), the possible major cause of enterocyte injury (Leelahavanichkul et al., 2016b) (**Table 1**), and gut barrier defect (Amornphimoltham et al., 2019), were similar between these phases of dengue infection. However, the plasma leakage in the critical phase might have more intestinal impacts than the febrile phase that is severe enough to cause intestinal dysbiosis as indicated here by a higher abundance of *Bacteroidaceae* and *Escheriachia* in blood bacteriome or the increased lactulose-digestible bacteria by others (Clausen & Mortensen, 1997) (LMER test interference). The direct intestinal immunofluorescent-based histopathology (Boonhai et al., 2021) or other methods of gut barrier evaluation are interesting to use. Nevertheless, an increase in the abundance of these Gram-negative bacteria in the intestine possibly elevates LPS in gut contents and induces a higher level of endotoxemia in severe dengue infection. Perhaps prominent intestinal hypoxia from plasma leakage in patients with the critical phase might be responsible for the selection of pathogens in the critical phase as most pathogens have some virulence factors that enhance their viability in the harsh microenvironments (Walther & Ewald, 2004).

Because both *Bacteroidaceae* (Martens et al., 2009) and *Escheriachia* (Lucas et al., 2017) are possible pathogens, the increase of these bacteria in the gut and their DNA in the blood might be a poor prognostic factor of dengue infection. Likewise, a decrease in *Bifidobacteriaceae*, the beneficial bacteria (Raethong et al., 2021), in dengue (febrile and critical phases) when compared with control also highlighted dengue-induced gut dysbiosis. Perhaps the dysbiosis is started from reduced *Bifidobacteriaceae* in the febrile phase followed by increased *Bacteroidaceae* and *Proteobacteria* in the more severe critical phase which might be another predictor for dengue severity. Although the sequential exploration in the several time-points in the same patients with the disease progression from febrile into critical stages is not performed here, data of blood bacteriome in COVID-19 patients with endotoxemia from gut barrier defects demonstrate the progressive reduction of Firmicutes (the possible beneficial bacteria) DNA in blood at day 3 and day 7 post-COVID-19 compared with the 1st day of the infection (Sirivongrangson et al., 2020). Hence, the progression of gut dysbiosis along with the disease severity is possible and the manipulation of the dysbiosis, using probiotics, might be beneficial. More studies on this topic in patients and mice are needed. Not only bacterial DNA (Kaewduangduen et al., 2022), but LPS and BG (Issara-Amphorn et al., 2018) in serum from dengue-induced leaky gut syndrome also induce pro-inflammatory effect and more profoundly activate monocytes (Wu et al., 2013) and NK cells (Poggi et al., 2019; Chapuy & Sarfati, 2020) compared with patients without gut barrier defect. Indeed, monocytes are important immune cells that recognize pathogen molecules in several inflammatory diseases in either sepsis (Aguilar-Briseño et al., 2020) or non-sepsis (Grip et al., 2007; Mukherjee et al., 2015; Weldon et al., 2015). Although both LPS and BG can stimulate monocytes (Chan et al., 2009; Kew et al., 2017), the simultaneous presence of LPS with BG synergistically activates inflammation (Issara-Amphorn et al., 2018) which might be similar to the condition of dengue-induced leaky gut. On the other hand, the chronic presence of LPS might induce endotoxin tolerance, a reduced LPS responses after the first dose of LPS (Ondee et al., 2019), partly through the interference of cellular energy status (Gillen et al., 2021), which might be associated with ineffective organismal control after viral infection (post-dengue secondary bacterial infection) (Sharma, 2020). Perhaps bacterial DNA, LPS, and BG in dengue patients with leaky gut affects non-classical monocytes and NK cells that alter the pathogenesis of dengue infection (**Figure 4**). We hypothesize that systemic inflammation (a similar serum IL-6 level in both febrile and critical phases; **Table 1**) (Leelahavanichkul et al., 2016b), together with the direct DENV infection in enterocytes (Kar et al., 2019) induces gut leakage and gut translocation of several pathogen molecules that facilitates systemic inflammation in both stages of the infection. Although serum IL-6 is a biomarker associated with the severity of bacterial sepsis (Calandra et al., 1991), the IL-6 level could not differentiate between dengue in febrile and critical phases. Hence, the systemic inflammation in both febrile and critical phases in patients with gut barrier defects might be more severe than the patients without leaky gut. On the other hand, dengue patients with acute febrile phase demonstrated increased peripheral blood non-classical monocytes (CD14^-^CD16^+^) and NK cells (CD56^+^CD16^-^) compared with those in the critical phase (**Table 2**) possibly resulting in better control of the disease severity by innate immunity in the febrile phase over the critical stage. Perhaps the expanded CD14^-^CD16^+^ monocyte subsets in the acute febrile phase are responsible for the effective viral control and disease recovery in the later phase (Narasimhan et al., 2019), leading to a lower disease progression rate during the febrile phase at 62% (five from eight cases) compared to 82% (19 from 23 cases) of those with the critical phase. Likewise, prominent NK cells (CD56^+^CD16^-^) in the febrile phase compared to the critical phase supported early NK cell responses against DENV (Green et al., 1999; Chau et al., 2008; Keawvichit et al., 2018). A lower rate of disease progression in the febrile phase is possibly due to an impaired NK cell activation during the acute phase of dengue infection (Chau et al., 2008). Although the total abundance of T cells may not represent DENV-specific responsive cells, the increase in the absolute CD4 and CD8 T cells in the febrile phase compared to the critical phase (**Table 2**) might be a better DENV control of patients in the febrile phase (less severe than critical phase). Taken together, non-classical monocytes, NK cells, and cytotoxic T cells (CD8) may have a key role in the control of the dengue virus in the early stage of patients with severe infection. Dysregulation and aberrant innate immune responses further lead to reduced viral control and severe dengue by inducing vascular leakage and excessive inflammation due to the high levels of inflammatory cytokines (Malavige et al., 2020).

For the clinical translation, the evaluation of gut barrier defects in patients with dengue and the strengthening gut integrity (probiotics, short-chain fatty acid, and/or zonulin alteration) (Seo et al., 2021; Hoilat et al., 2022), LPS absorption (Lipcsey et al., 2020), and BG removal (Ohata et al., 2003) might be the interesting adjuvant therapies that attenuate the disease mortality. More studies are warranted. There were several limitations in our study, including the limited parameters of innate immune analyses (cannot rule out the effect of other cells against dengue). Due to the cross-sectional nature of the analysis with a limited number of patients and blood microbiome evaluation, studies in more time points might be informative. Next, the recruitment of only dengue patients with intestinal permeability that were detected using oral administration-dependent lactulose-to-mannitol excretion ratio (LMER). Then, the very severe dengue infection (shock or hemorrhage) could not be enrolled (unable to do the LMER test). Finally, the study was not directly designed for the prediction of clinical outcomes, although we hypothesized that both innate and adaptive immune parameters could help determine fatal outcomes. Further studies on the topic are needed.

In summary, leaky gut and gut translocation of pathogen molecules might cause sepsis in dengue infection through the activation of non-classical monocytes and NK cells. Blood bacteriome and the manipulations on gut dysbiosis might be useful for biomarker or adjuvant therapy of dengue-induced leaky gut. The increased understanding of immune responses in dengue infection is likely to move toward the rational development and the use of novel approaches for disease modulation in the near future.

## Data Availability Statement

The datasets presented in this study can be found in online repositories. The name of the repository and link to the data can be found below: NCBI; PRJNA838435.

## Ethics Statement

The study was approved by the Ethics Committee of the Faculty of Tropical Medicine, Mahidol University (MUTM 2020-071-01). The patients/participants provided their written informed consent to participate in this study.

## Author Contributions

Conceptualization, WC, SK, WA, and AL; methodology, WC and SK; validation, WC and WA; formal analysis, WC and AL; investigation, WC and AL; resources, WC and WA; data curation, WC and WA; writing—original draft preparation, WC, SK, and AL; writing—review and editing, WC, AL, VT, WP, and PW; visualization, WC, AL, WP, and PW; supervision, AL, VT, WP, and PW; project administration, WC and SK; funding acquisition, WC. All authors have read and agreed to the published version of the manuscript.

## Funding

This work was funded by Mahidol University–Basic Research Fund: fiscal year 2021 (FRB640032) (Contract No. BRF1-A42/2564) (WC).

## Conflict of Interest

The authors declare that the research was conducted in the absence of any commercial or financial relationships that could be construed as a potential conflict of interest.

## Publisher’s Note

All claims expressed in this article are solely those of the authors and do not necessarily represent those of their affiliated organizations, or those of the publisher, the editors and the reviewers. Any product that may be evaluated in this article, or claim that may be made by its manufacturer, is not guaranteed or endorsed by the publisher.
